# From two to one: resolving CO binding in acetyl-CoA synthase

**DOI:** 10.1039/d5sc08875e

**Published:** 2026-01-29

**Authors:** Denise Poire, Cornelius C. M. Bernitzky, Mathesh Vaithiyanathan, Berta M. Martins, Christian Lorent, Tamanna M. Ahamad, Vladimir Pelmenschikov, Igor Sazanovich, Gregory M. Greetham, Ingo Zebger, Holger Dobbek, Maria Andrea Mroginski, Marius Horch

**Affiliations:** a Freie Universität Berlin, Department of Physics, Ultrafast Dynamics in Catalysis Arnimallee 14 14195 Berlin Germany marius.horch@fu-berlin.de; b Technische Universität Berlin, Department of Chemistry, Modelling of Biomolecular Systems Straße des 17. Juni 135 10623 Berlin Germany; c Humboldt-Universität zu Berlin, Department of Biology, Structural Biology/Biochemistry Unter den Linden 6 10099 Berlin Germany; d Technische Universität Berlin, Department of Chemistry, Spectroscopic Characterization of Metalloproteins Straße des 17. Juni 135 10623 Berlin Germany; e STFC Central Laser Facility, Research Complex at Harwell, Rutherford Appleton Laboratory, Harwell Campus Didcot OX11 0QX UK

## Abstract

Acetyl-CoA synthase (ACS) catalyzes the condensation of acetyl-CoA from carbon monoxide (CO), a methyl group, and coenzyme A, enabling the fixation of CO into biomolecules. Recent low-temperature ENDOR studies proposed that the enzyme can bind two CO ligands in its reduced A_red_–CO state, reshaping the view of CO coordination and inhibition of ACS. However, whether this two-CO model reflects a physiologically relevant state has remained an open question. To address this issue, we examined ACS under near-native, ambient conditions using ultrafast and two-dimensional infrared spectroscopy, complemented by anharmonic frequency calculations. These methods provide a wealth of structural and dynamical information beyond insights from conventional IR absorption spectroscopy, allowing a direct view of CO coordination in the A_red_–CO state. Our results demonstrate that ACS binds a single CO ligand under ambient conditions. This finding clarifies the stoichiometry of CO coordination in ACS and underscores the broader potential of advanced IR spectroscopy, combined with computation, to unravel ligand binding in complex bioorganometallic systems.

## Abstract 2

Acetyl-CoA synthase is a key enzyme in biological carbon fixation. Combining ultrafast and two-dimensional infrared spectroscopy with anharmonic frequency calculations, we demonstrate that the enzyme binds a single CO ligand under native conditions.

## Graphical abstract

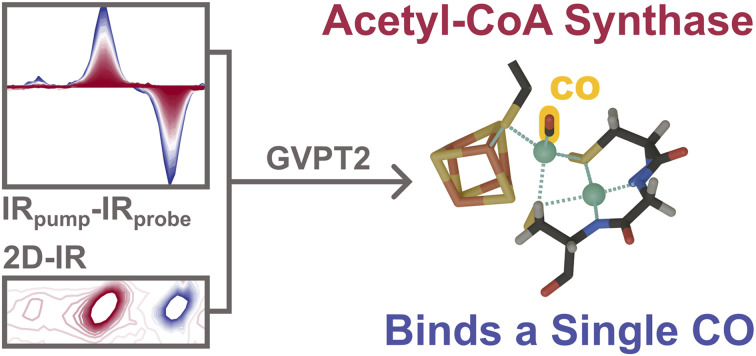

## Introduction

**Fig. 1:**
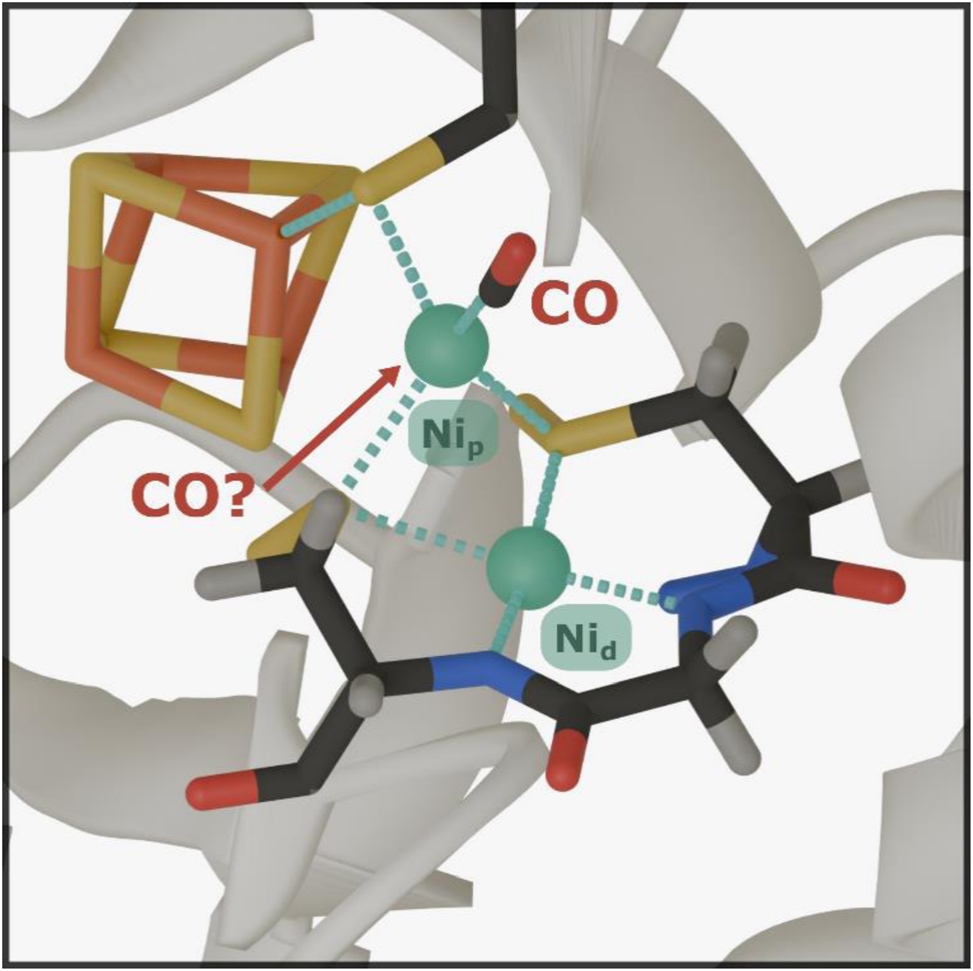
Crystal-structure representation of the ACS A-cluster in the A_red_–CO state. The shown structure corresponds to the enzyme from *C. hydrogenoformans* (PDB ID: 7NYS). The A-cluster consists of a di-nickel center that is covalently linked to a cubane [4Fe4S] cluster *via* a cysteine ligand coordinated to the so-called proximal nickel (Ni_p_). Ni_p_ is linked, through two additional cysteine sulfur atoms, to the distal nickel (Ni_d_), which is fixed in a square planar arrangement by coordination through the protein backbone and the cysteine side chains. Critical to the A_red_–CO state is the redox-active Ni_p_, which is coordinated by a CO ligand. The putative binding site for a second CO ligand is marked by an arrow. Color scheme: iron in orange, sulfur in yellow, nickel in green, carbon in dark gray, oxygen in red, nitrogen in blue, and hydrogen in light gray.

Acetyl-CoA synthase (ACS) is a crucial enzyme that catalyses the formation of acetyl-CoA from carbon monoxide (CO), a methyl group, and coenzyme A (CoA).^1,2^ As such, it plays a central role in the ancient Wood-Ljungdahl Pathway (WLP).^1^ This pathway is a highly efficient carbon fixation process utilized by certain anaerobic microorganisms to reduce carbon dioxide (CO_2_) for incorporation into organic compounds, thereby making a significant contribution to the global carbon cycle. Investigating the structural and dynamical properties of ACS is essential for understanding the key determinants of biological CO transfer. Such insights hold the promise of revealing sustainable routes for carbon sequestration and the efficient production of valuable C2 compounds, addressing critical environmental challenges while inspiring innovative industrial applications for CO utilization.^2–4^ The active site of ACS (Fig. 1), known as the A-cluster, is a remarkable catalytic site featuring a canonical [4Fe4S] cluster linked by a cysteine sulfur atom to a unique binuclear centre composed of two nickel (Ni) ions. The proximal nickel close to the [4Fe4S] cluster, Ni_p_, is the redox-active site where both carbonylation and methylation are proposed to occur, while the distal nickel, Ni_d_, remains in a square planar Ni^2+^ state throughout the catalytic cycle.^2–6^ The carbonylated state of the A-cluster (A_red_–CO), characterized by a Ni_p_^1+^–CO motif bound to Ni_d_^2+^, has been extensively studied.

A recent study using electron paramagnetic resonance (EPR) and electron nuclear double resonance (ENDOR) techniques has suggested that the A-cluster in ACS may bind two CO molecules.^7^ One CO, whose binding configuration has already been characterized by crystallography,^5^ binds to the axial position of the *d*_z_^2^ orbital of Ni_p_. The additional CO was hypothesized to bind reversibly in the equatorial plane, occupying the position typically held by the methyl group. However, this proposed second binding site has only been reported based on studies at cryogenic temperatures (2 K), as dictated by the employed techniques. Determining whether ACS can bind two CO molecules under biologically relevant conditions and ambient temperatures is crucial, as it could significantly reshape our understanding of the enzyme's catalytic mechanism and regulation. For instance, the formation of a complex with two CO molecules bound could explain the observed inhibition of ACS at high CO concentrations or serve as a structural analogue for a ‘two-substrate-bound’ state that has both the methyl group and the CO coordinated to Ni_p_ before C–C bond formation.^7–9^

To explore the hypothesis of dual CO binding in ACS, we employed advanced nonlinear infrared (IR) spectroscopic techniques, complemented by computational methods. One of the main advantages of both linear and nonlinear IR spectroscopy is the ability to investigate proteins under near native conditions. In addition, IR spectroscopy is particularly suited for studying ligand binding in (bio)organometallic compounds, *e.g.* CO adducts of metalloproteins (such as CO-bound hemoproteins) or hydrogenases.^10–24^ First, the fundamental CO stretching frequency and other spectroscopic observables related to this bond-localized oscillator are highly sensitive to changes in metal–CO and C≡O bonding as well as the (electronic) structure of the coordinated metal site as a whole. Second, CO exhibits a considerable transition dipole moment (IR activity), which is further enhanced upon metal binding. Finally, the fundamental CO stretching frequency appears in a distinct region of the IR spectrum, free from other contributions of the protein. This selective spectral window allows for a direct, sensitive probing of bioorganometallic targets, making IR spectroscopy a valuable tool for characterizing the carbonylated A_red_–CO state of ACS.^11,25^

Despite its merits, linear IR absorption spectroscopy faces fundamental limitations, particularly in terms of resolving overlapping signals, revealing details of the molecular and vibrational structure, and extracting dynamic information. In contrast, ultrafast nonlinear IR techniques provide insights into these aspects with very high time resolution. Unlike linear IR spectroscopy, which relies on the single interaction of a continuous IR beam, nonlinear IR methods leverage multiple sequential interactions of ultrashort IR laser pulses with the sample, inducing a series of vibrational transitions that reflect not only molecular structure but also its time evolution. As a result, these techniques can reveal intricate details unavailable from conventional IR spectroscopy. In this study, we utilized nonlinear IR_pump_–IR_probe_ spectroscopy and two-dimensional infrared (2D-IR) spectroscopy. In an IR_pump_–IR_probe_ experiment, an IR pump pulse vibrationally excites the sample, and an IR probe pulse tracks the resulting changes in absorption as a function of frequency. Expanding this concept, 2D-IR spectroscopy introduces an additional IR pump pulse to resolve the IR_pump_–IR_probe_ spectrum with respect to the pump frequency.^26–29^ The result is a two-dimensional spectrum that correlates excitation (pump) and detection (probe) frequencies.^30^

To complement the experimental data, we employed generalized second order vibrational perturbation theory (GVPT2)^31–35^ to compute key observables from nonlinear IR spectra beyond the harmonic limit. Since the signals observed in nonlinear IR spectroscopy are a direct consequence of the anharmonicity of the potential energy surface (PES), anharmonic frequency calculations are indispensable, and (G)VPT2 represents the only suitable approach that can be applied to systems with more than a few atoms.

By employing these advanced nonlinear IR techniques and complementary computational tools, we have gained detailed insights into the structural and dynamical properties of the A_red_–CO state of ACS, utilizing the enzyme from *Carboxydothermus hydrogenoformans* as a model system. Our results demonstrate that, at ambient temperature and under near-native conditions, the ACS active site binds a single CO ligand.

## Results and discussion

**Fig. 2:**
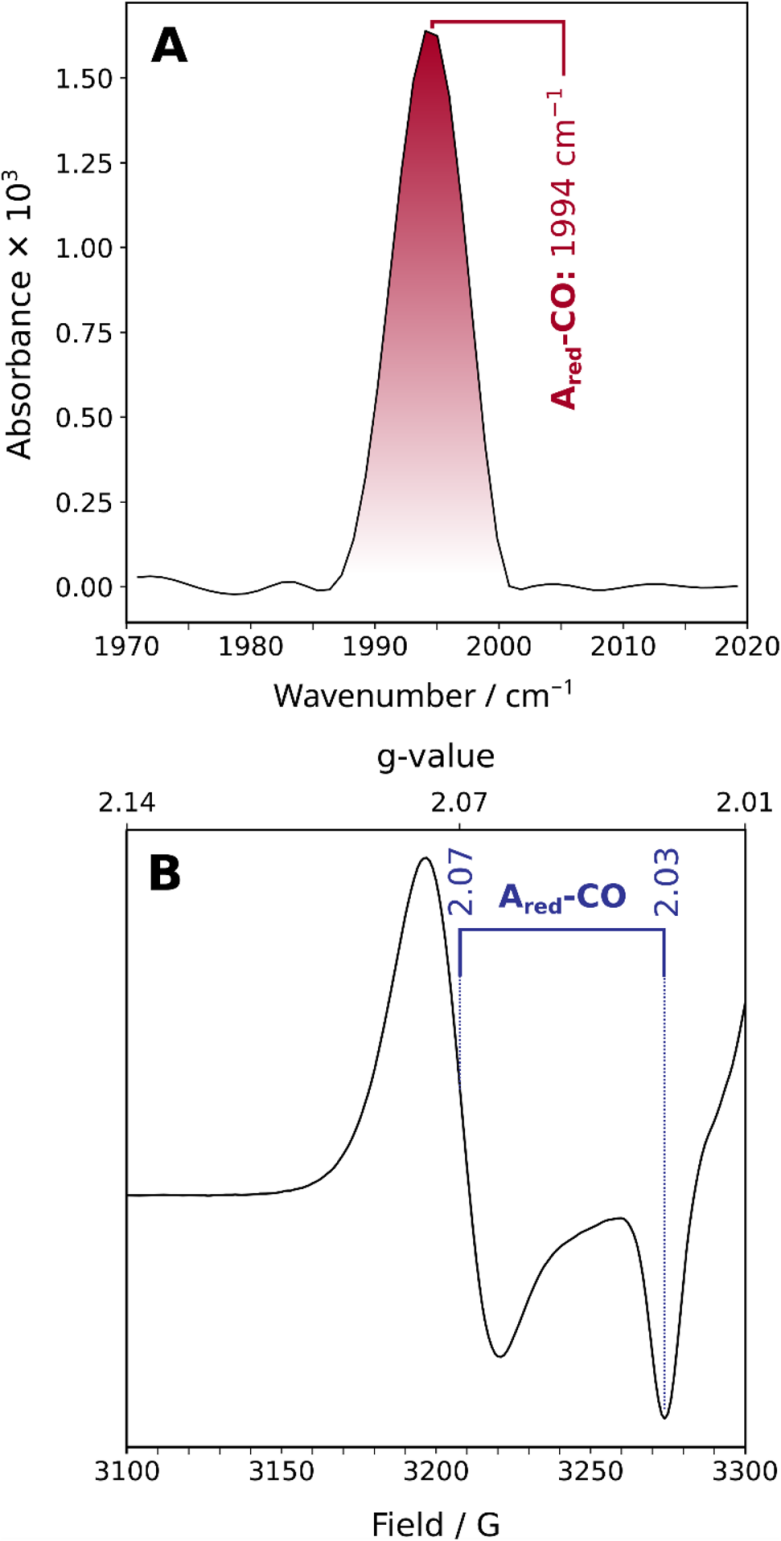
Linear IR absorption spectrum (A) and X-band EPR spectrum (B) of the A_red_–CO state of ACS from *C. hydrogenoformans*. The IR spectrum was recorded at 283 K, and the EPR spectrum was recorded at 80 K.

The ambient-temperature IR absorption spectrum of reduced and CO-incubated ACS from *C. hydrogenoformans* (Fig. 2A and S1A), recorded at 283 K, shows a distinct peak at *ca.* 1994 cm^−1^, corresponding to the stretching vibration of a CO molecule bound to the Ni site (the reported frequency varies between 1993 and 1996 cm^−1^). This confirms the presence of a CO molecule bound to the active site, as previously noted.^11,25^ The EPR spectrum recorded at 80 K also indicates that the active site resides in a reduced state with a mixed-valent Ni_d_^2+^Ni_p_^1+^ ground state (Fig. 2B and S1B). Thus, the combination of IR and EPR results confirms that the active site is in the previously studied A_red_–CO state, with a bound CO molecule and the Ni_p_ in the +1 oxidation state. This observation demonstrates that the enzyme samples studied here are in the same state as those investigated previously.^6,11^

While the linear IR spectrum indicates the presence of a single CO ligand bound to the active site of ACS in the A_red_–CO state at ambient temperature, the presence of a second CO ligand, as proposed in a recent ENDOR study,^7^ cannot be categorically ruled out for two reasons. (1) In general, two CO ligands bound to a single metal should give rise to two distinct and coupled modes, *i.e.* symmetric and antisymmetric combinations, or – less likely – two bond-localized modes if the two CO ligands are chemically very different. In both cases, the two modes would feature different frequencies. Alternatively, the two putative CO ligands might – at least hypothetically – be chemically indistinguishable but uncoupled, thereby leading to two localized and energetically near-degenerate CO stretching modes. (2) One of the two (coupled) CO stretching modes may feature a significantly lower transitions dipole moment than the other, thereby becoming essentially IR-inactive.

To explore these possible scenarios, we employed advanced nonlinear IR techniques together with complementary computational tools. We start our analysis using ultrafast IR_pump_–IR_probe_ spectroscopy. IR_pump_–IR_probe_ spectra are displayed as difference spectra, which illustrate the changes in IR probe light absorption that occur after the sample has been perturbed by the IR pump pulse.

**Fig. 3:**
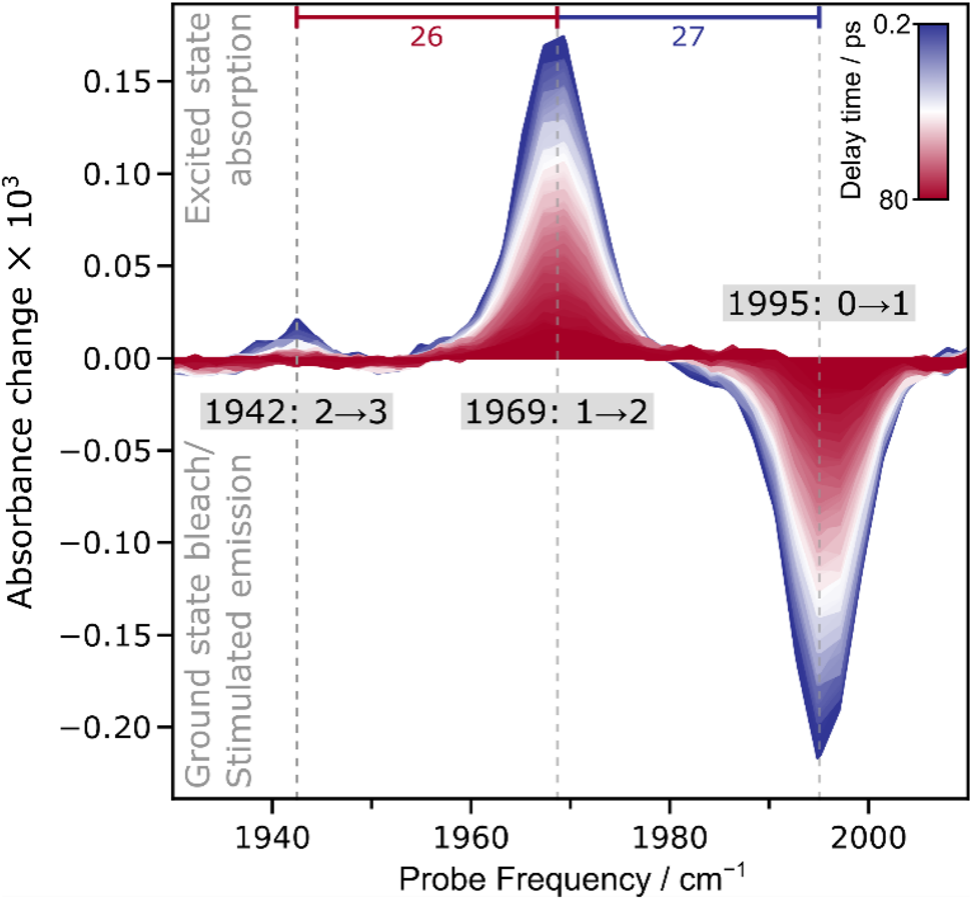
Time series of IR_pump_–IR_probe_ spectra recorded from the A_red_–CO state of ACS from *C. hydrogenoformans*. Spectra were acquired at 283 K with parallel polarization of pump and probe pulses. The anharmonicities *E*(0 → 1) – *E*(1 → 2) and *E*(1 → 2) – *E*(2 → 3) are indicated as blue and red numbers, respectively.

In the ambient-temperature IR_pump_–IR_probe_ spectrum, recorded at 283 K, (Fig. 3), a single negative feature and a sequence of positive signals can be observed. The negative feature at 1995 cm^−1^ is assigned to the bleaching of the vibrational ground state (*v* = 0) and stimulated emission from first excited state (*v* = 1) of the CO stretching vibrational mode. The observation of a single signal at the fundamental absorption frequency *E*(0 → 1) supports the presence of a single CO ligand bound to the active site of ACS in the A_red_–CO state. Positive signals in the IR_pump_–IR_probe_ spectrum can be assigned to excited-state absorption, *i.e.* transitions between higher vibration levels, denoted as *v* → *v* + 1. Absorption of the first excited state (1 → 2) yields a signal at 1969 cm^−1^, while absorption of the second excited state (2 → 3) is observed at 1942 cm^−1^.

Visual inspection of both linear IR absorption and nonlinear IR_pump_–IR_probe_ spectra indicates the presence of a single CO ligand bound to the active site of ACS in the A_red_–CO state. To evaluate the possible presence of overlapping signals that could obscure closely spaced peaks, potentially corresponding to two CO molecules bound to the metal, we performed a lineshape analysis on both the linear IR absorption spectrum and the IR_pump_–IR_probe_ spectra. For the linear IR spectrum, the fitting was performed using both single and double Gaussian lineshape models. In the single Gaussian fit, the peak position was identified as 1994.31 cm^−1^ (see Fig. S2 and Table S1). When a second Gaussian component was introduced, its amplitude was nearly zero (10^−6^), effectively collapsing the fit back to a single Gaussian at 1994.31 cm^−1^ (see Fig. S2 and Table S1). In addition, the *R*^2^ values for the two models were very similar, with 0.9965 for the single Gaussian and 0.9973 for the double Gaussian, indicating only a slight improvement with the addition of a second (negligible) component. Moreover, when we evaluated the Akaike Information Criterion (AIC) and Bayesian Information Criterion (BIC), the single Gaussian model showed lower values compared to the double Gaussian model, indicating that it provided a better overall balance between fit quality and model complexity. A similar fitting approach was applied to the IR_pump_–IR_probe_ spectra, analysing 0 → 1 and 1 → 2 transitions, both of which exhibit appreciable intensity (Fig. S3, S4, and Tables S2–S4). For this analysis, two approaches were used. On the one hand, the spectra were averaged across all time delays, and both single and double Gaussian models were compared. As with the linear IR data, the *R*^2^ values for the single and double Gaussian fits were nearly identical, showing no significant improvement with the addition of a second Gaussian component. Once again, the AIC and BIC values were lower for the single Gaussian model, confirming that it offered a more balanced model. On the other hand, when only the most intense spectra at early pump-probe delay times (10 spectra between 0.199 ps and 2.089 ps) were analysed, no convergence was observed for the double-Gaussian fit, indicating that any second component represents an artefact. In total, the analysis of both the linear IR absorption spectrum and the IR_pump_–IR_probe_ spectra supports the conclusion that the lineshapes of the vibrational transitions are best described by a single Gaussian, with no evidence of signal overlap.

Due to the observation of excited-state absorption signals in the IR_pump_–IR_probe_ spectrum (*vide supra*), we can extract the anharmonicity associated with the CO stretch vibrational potential. The anharmonicity, defined here as the energy difference between subsequent vibrational transitions, reflects the deviation of the potential energy surface along the probed vibrational coordinate from the parabolic shape expected for an idealized harmonic oscillator. For diatomic ligands bound to metal sites, the anharmonicity is an indicator for mode localization, *i.e.* the magnitude of this value is sensitive to the ligand stoichiometry. In the current case, the anharmonicity is nearly constant, *i.e.* 27 cm^−1^ for *E*(0 → 1) – *E*(1 → 2) and 26 cm^−1^ for *E*(1 → 2) – *E*(2 → 3). In fact, the two values are identical within the fit error (see Fig. S4 and Table S4), a behaviour that fits to an idealized Morse oscillator, *i.e.* the bond-localized vibration of a chemical group that can be approximated as a single diatomic molecule. In addition, the observed anharmonicity values fit to literature reports of monocarbonyl compounds, while mononuclear dicarbonyl species would yield much smaller values for the resulting symmetric and antisymmetric modes.^36–45^

**Fig. 4:**
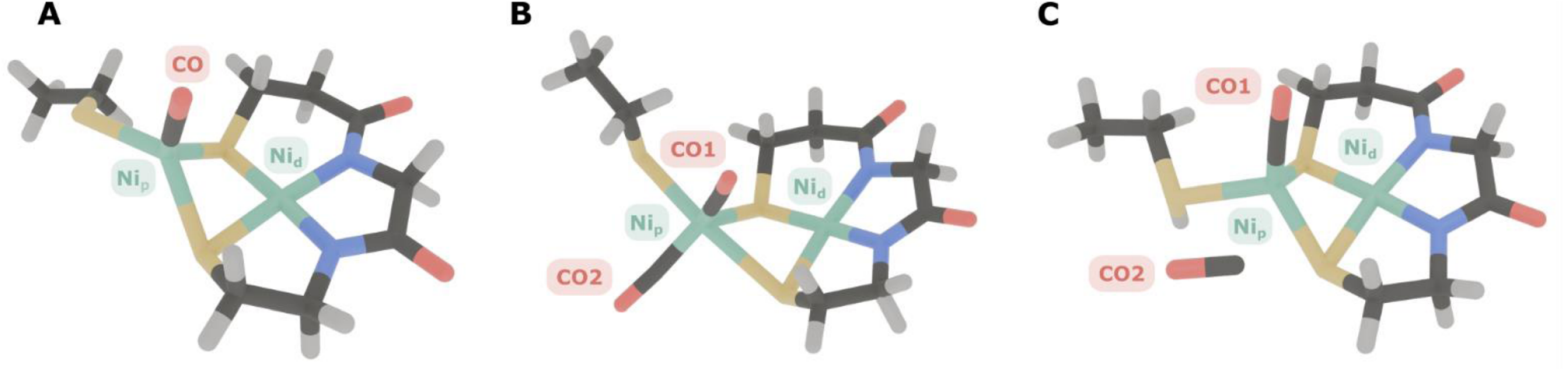
Optimized geometries of ACS active-site models used in anharmonic vibrational analyses. (A) Monocarbonyl model with a single CO ligand axially bound to Ni_p_. (B) Dicarbonyl model featuring two CO ligands bound to Ni_p_. (C) Non-coordinated model with a second CO placed near the active site but not directly coordinated to Ni_p_. Color scheme: sulfur in yellow, nickel in green, carbon in dark gray, oxygen in red, nitrogen in blue, hydrogen in light gray.

To illustrate the difference in anharmonicities of mono- and dicarbonyl species, we conducted an anharmonic vibrational analysis on the density functional theory level using an automatized GVPT2 approach.^31–35^ Two computational models of the A-cluster were initially investigated: one with a single CO molecule bound and another one with two CO molecules in the configuration proposed by the above-mentioned ENDOR study.^7^ For the two-CO model (Fig. 4B), the calculated anharmonic frequencies for the two normal modes predominantly corresponding to the symmetric (in-phase) and antisymmetric (out-of-phase) combinations of the two CO ligands, were 1893 and 1924 cm^−1^, respectively. For the single-CO model (Fig. 4A), the CO stretching mode had an anharmonic frequency of 1881 cm^−1^. While these absolute vibrational frequencies are very sensitive to the level of theory and other aspects of the computational approach, the anharmonicities (transition energy differences) that reflect the shape of the PES and the ligand stoichiometry can be calculated with high accuracy.^46^ In the case of the single-CO model, the calculated intramode anharmonicity was 25 cm^−1^, closely matching general expectations^38,44,46^ and the values observed in our experimental spectra (see Table 1). By contrast, the two-CO model exhibited significantly lower anharmonicities, with values of 13 cm^−1^ and 16 cm^−1^, which are reasonable for dicarbonyl compounds^40–51^ but inconsistent with the experimental data.

**Table 1:** Comparison of experimental and theoretical anharmonicities for different ACS models^a^

Species	Anharmonicity/cm^−1^	
	CO1	CO2
Model A	25	—
Model B	13	16
Model C	24	32
Experimental value	27	

a Computational models are labelled according to Fig. 4. CO1 is coordinated to Ni_p_ in all computational models. CO2 is coordinated to Ni_p_ in Model B and non-coordinated in Model C. The experimental anharmonicity reflects the transition energy difference *E*(0 → 1) – *E*(1 → 2).

These findings highlight five key points: (I) The monocarbonyl model aligns well with the experimental data. (II) In a mononuclear dicarbonyl species of ACS, the two coupled modes must have different frequencies, ruling out the possibility of degenerate, bond-localized vibrations. (III) The diagonal (intramode) anharmonicities of the coupled modes are significantly reduced compared to the monocarbonyl model, which further indicates an inconsistency of the dicarbonyl model with the experimental data. (IV) Even if the fundamental frequencies of two putative CO modes were nearly identical, excited-state absorption frequencies would still be distinguishable due to the significantly different anharmonicities. Experimental excited-state absorption signals are inconsistent with such a scenario, though, ruling out a second CO ligand bound to ACS in the A_red_–CO state. (V) Two CO ligands, if present, would be coupled, thereby giving rise to two CO stretch modes with a fundamental splitting on the order of 30 cm^−1^. This finding rules out the recent claim that a weak feature, previously observed at 2044 cm^−1^,^47^ reflects the presence of a second CO ligand.^7^ First, if that was the case, the fundamental splitting would be 50 cm^−1^, *i.e.* unusually large and incompatible with the calculations. Second, the presence of the 2044 cm^−1^ signal has no noticeable effect on frequency of the canonical CO stretch mode at 1994 cm^−1^, in sharp contrast to expectations for two modes arising from coupled CO ligands. We therefore propose that the 2044 cm^−1^ signal reflects a different state (or degradation product) of the enzyme with lower backbonding, indicative of coordination to Ni^2+^ rather than Ni^1+^.

An additional scenario to consider is the presence of a second CO molecule that is not coordinated to Ni but residing in close proximity to the active site (Fig. 4C). This non-coordinated CO might exhibit a significantly smaller transition dipole moment, thereby becoming essentially IR-inactive. In addition, it could be uncoupled from the Ni-coordinated CO, so that both CO stretching vibrations would exhibit anharmonicities typical for monocarbonyl species. Our calculations show that, even in such a dissociated arrangement, the unbound CO would still interact with the Ni-bound CO through transition dipole coupling (TDC). This coupling would impart enough IR intensity for the dissociated CO to be detectable (intensity ratio of approximately 0.53 relative to the Ni-bound CO).

Notably, the reported intensity ratio reflects a low limit for such a scenario since the distance and relevant angles associated with the two COs are quite unsuitable for efficient TDC. TDC depends crucially on the distance and relative orientation of the dipoles, being most effective when the dipoles are nearly collinear and separated by a short distance.^48^ In our model (Fig. 4C), however, the CO–CO distance (*ca.* 3.26 Å) and the near-perpendicular arrangement (∠C1–O1–C2–O2 = −91.6°) render the geometry unfavourable for efficient coupling. The same is true for the angles between the two CO bond coordinates and the line connecting their geometric centres (77.60° and 109.25° for the coordinated and non-coordinated CO, respectively). Hence, if a second, unbound CO was present near the ACS active site, it should appear as a separate feature in the IR spectrum or cause notable broadening in the observed signals. Since no such additional band or broadening is observed in our linear and nonlinear IR spectra, we can rule out the possibility of a second, dissociated CO near the active site. This statement is also supported by differences in fundamental frequencies (1946 *vs.* 2055 cm^−1^) and anharmonicities of the two CO modes. Interestingly, the anharmonic vibrational analysis for this model yields anharmonicities of about 24 cm^−1^ for the Ni-bound CO and 32 cm^−1^ for the non-coordinated CO. The latter value is extremely high but consistent with some experimental reports on CO photolyzed from metal sites and located inside protein cavities.^20^ Reproduction of such observations by a first-coordination-sphere computational model indicates that high anharmonicities of non-coordinated CO are not necessarily dictated by the dielectric environment of the protein cavity but by electrostatic interactions with the nearby metal site and/or the ligands. We propose that the high anharmonicity reflects a decrease in bond dissociation energy due to stabilization of the negative partial charge of the carbon atom. Further analyses are necessary to understand the fundamental impact of this phenomenon on bonding and non-bonding interactions in metal carbonyl compounds.

**Fig. 5:**
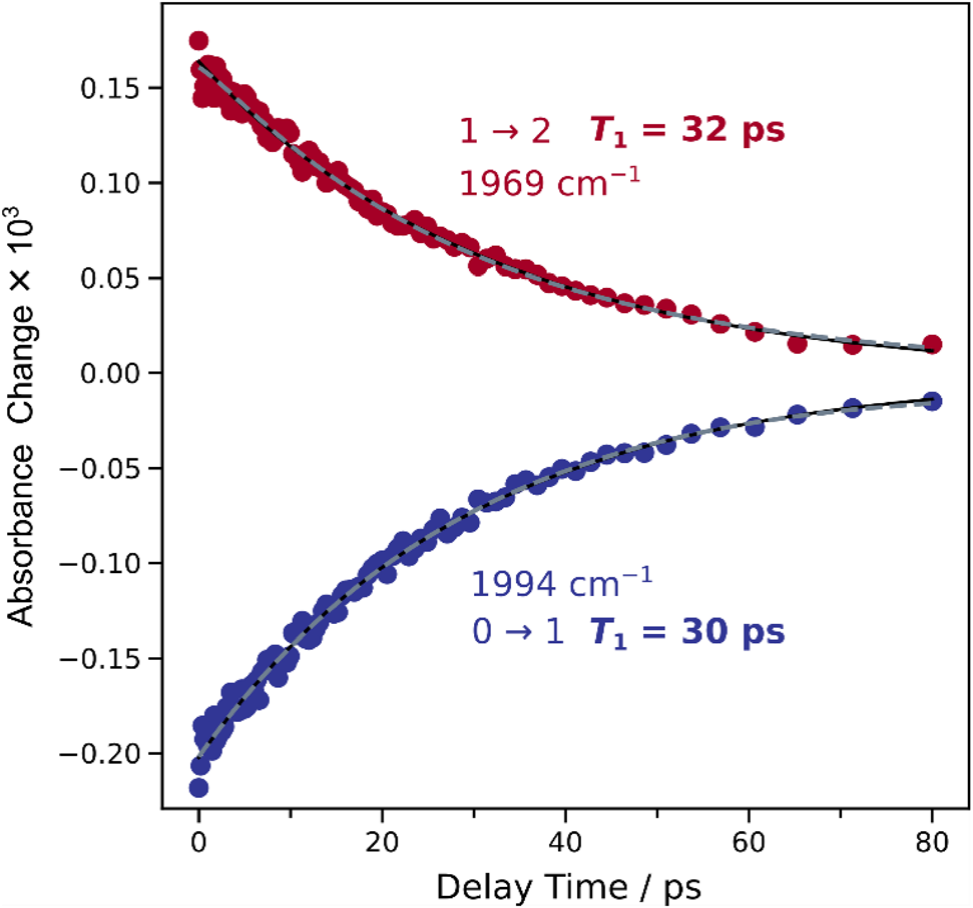
Time evolution of signal intensities extracted from the time series of the IR_pump_–IR_probe_ spectra shown in Fig. 3. Blue and red datapoints reflect signal intensities at *ca.* 1995 cm^−1^ (0 → 1) and 1969 cm^−1^ (1 → 2), respectively. Vibrational lifetimes, *T*_1_, obtained by a monoexponential fit (black line) are indicated. A biexponential fit (dashed grey line) is shown for comparison. Fit metrics and results are summarized in Tables S5 and S6, respectively.

After analysing the vibrational structure of A_red_–CO, we next turn to dynamical observables that can be extracted from the IR_pump_–IR_probe_ spectra. The signal amplitudes in these spectra decay as a function of the pump-probe delay time, due to relaxation of the vibrationally excited states. The associated time constants are the (apparent) vibrational lifetimes of these states that reflect energy transfer from the probed vibrational mode towards the (protein) environment. If there were two near-degenerate (uncoupled) CO stretching modes hidden in the envelope of the single set of signals (reflecting two CO ligands), two distinct vibrational lifetimes should be extractable from the decay curves. To investigate this aspect, a comparative analysis of mono- and biexponential fit models was conducted (Fig. 5). A single exponential decay adequately describes the temporal evolution of both transitions, while the biexponential fit does not provide a statistically significant improvement. Fitting metrics comparing mono- and biexponential fits are provided in the SI (Table S5), along with extracted vibrational lifetimes (*T*_1_) and corresponding amplitudes (Table S6). This result suggests either the presence of a single metal-bound CO molecule or multiple CO molecules with identical vibrational lifetimes. The latter option can be essentially excluded since the two putative CO ligands would necessarily face different protein environments, and their bonding to the central Ni ion cannot be identical within the low-symmetry architecture of the ACS active site.

While the presence of two coupled vibrational modes was essentially excluded by the analysis of the observed anharmonicities (*vide supra*), we further explored this possibility by analysing IR_pump_–IR_probe_ decay curves for indications of anharmonic coupling. In the presence of anharmonic coupling, selected Liouville pathways of a third-order nonlinear experiment lead to interstate coherences that manifest as sinusoidal patterns in the time evolution of IR_pump_–IR_probe_ spectra.^30^ Such patterns, also called quantum beats, were previously observed for dicarbonyl and dicyanido metal compounds,^36,37,49^ and their detection was utilized to indirectly probe IR-inactive vibrational modes.^37^ To assess this possibility, a simple monoexponential decay model was compared to a more complex one incorporating both an exponential decay and a damped sinusoid (Fig. S5). An F-test yielded a *p*-value greater than 0.05, indicating no statistical preference for the oscillatory model (Table S7). Residual analysis of the monoexponential fit revealed no significant periodic structure, a conclusion also supported by Fourier transform analysis (Fig. S5), which showed no prominent peaks in the resulting quantum-beat spectrum. These findings suggest the absence of quantum beats, supporting the conclusion that the ACS active site binds a single CO molecule, as two CO molecules would likely produce two coupled modes and observable beating.

To further investigate the potential binding of one or two CO molecules to the ACS active site, 2D-IR spectroscopy was employed. By spreading the information from IR_pump_–IR_probe_ vibrational spectra over two dimensions, 2D-IR spectroscopy independently measures both excitation and detection frequencies, enabling the identification of cross-peaks that directly reflect vibrational coupling between modes, even in cases where one of the vibrational modes is not directly observable due to a small transition dipole moment. This dual–frequency correlation also facilitates the deconvolution of overlapping signals, allowing for a better separation of closely spaced vibrational modes.

Similarly to the IR_pump_–IR_probe_ experiments, the 2D-IR spectra allowed measuring the anharmonicity of the CO vibrational mode. The anharmonicity values were found to be 27 cm^−1^ for *E*(0 → 1) – *E*(1 → 2) and 26 cm^−1^ for *E*(1 → 2) – *E*(2 → 3), consistent with the values obtained from the IR_pump_–IR_probe_ data, additionally supporting the conclusion of a bond-localized CO stretching mode associated with a single metal-bound CO ligand. The extracted peak positions and anharmonicity values from the Gaussian fitting are provided in Fig. S6 and Table S8.

**Fig. 6:**
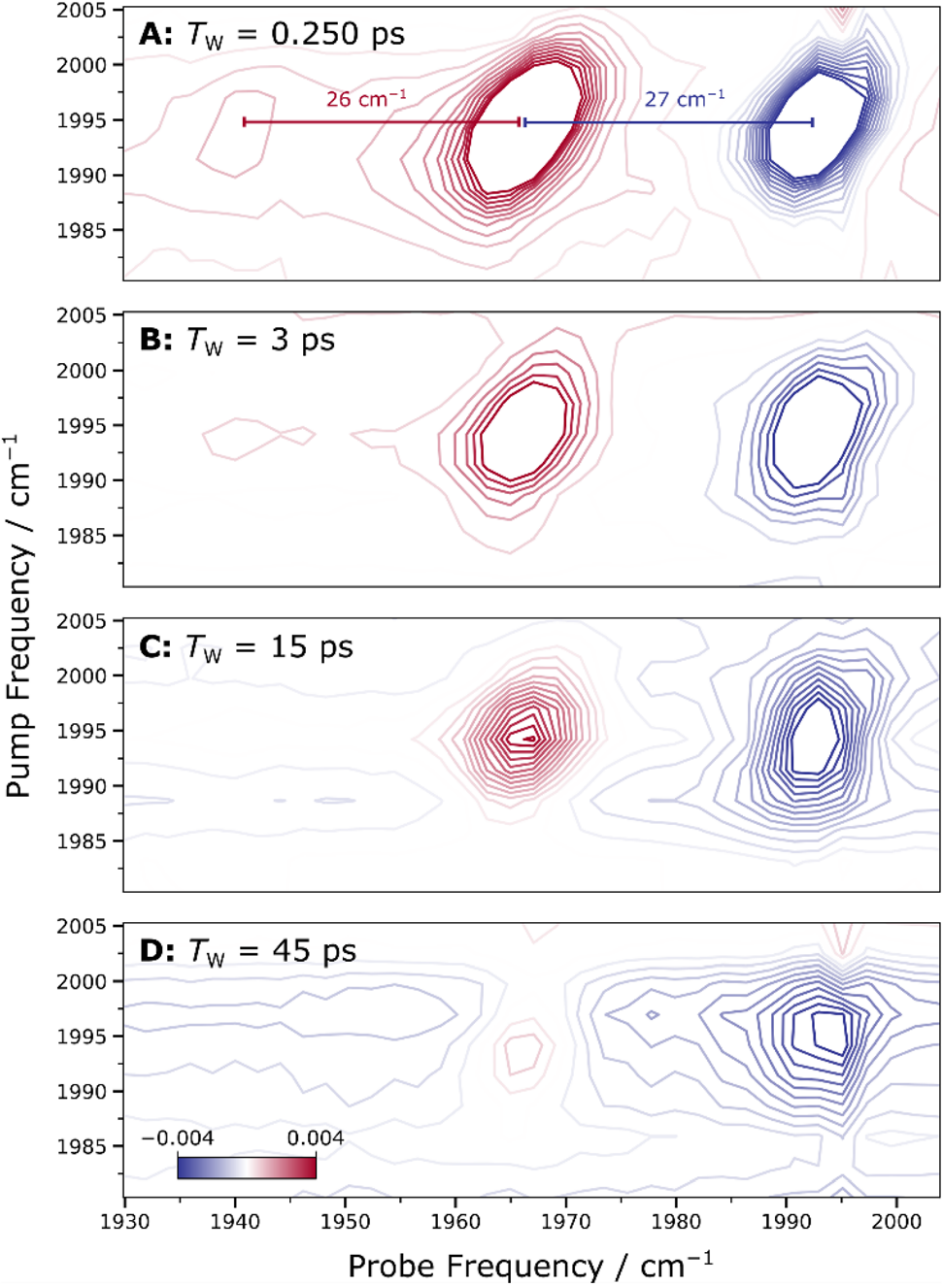
Time evolution of 2D-IR spectra recorded at different waiting times *T*_w_, as indicated. Panels A–D illustrate the time evolution of the spectroscopic lineshape (see Table 2 for a quantitative analysis of diagonal and anti-diagonal widths). In Panel A (*T*_W_ = 0.250 ps), the extracted anharmonicities of 27 cm^−1^ for *E*(0 → 1) – *E*(1 → 2) and 26 cm^−1^ for *E*(1 → 2) – *E*(2 → 3) are indicated by the red and blue markers, respectively (see Fig. S6 and Table S8 for fit details). Spectra were acquired at 283 K with parallel polarization of pump and probe pulses.

The possibility of two CO molecules being present was further investigated by examining the 2D-IR spectra for cross-peaks (Fig. 6), which would indicate vibrational coupling between two CO stretch vibrational modes and reveal dark transitions *via* coupling to bright modes. However, no distinct cross-peaks were detected. If two putative CO stretch modes had near-degenerate frequencies, the associated cross-peaks could be overlapped with the diagonal signals, blending into a more complex, potentially square-shaped pattern deviating from the typical elliptical shape. The absence of such a pattern suggests that if any cross-peaks were present, they would be either undetectable or overshadowed by the stronger diagonal signal. To explore this possibility, we conducted additional analyses.

First, the number of contour levels was increased to enhance the visibility of low-intensity features (Fig. S7). However, rather than revealing cross-peaks or a square-shaped pattern indicative of coupled CO modes, the spectra showed elongation along the diagonal, which aligns with inhomogeneous broadening. We then analysed horizontal slices of the 2D-IR spectrum at pump frequencies both higher and lower than the diagonal maximum (Fig. S8). These horizontal slices showed no strong off-diagonal features, further suggesting the absence of vibrational coupling between two putative CO modes, as the signal remained localized along the diagonal. Finally, anti-diagonal slices were examined to identify any subtle broadening patterns or secondary features (Fig. S9). These slices revealed single, smooth peaks without any observable secondary features, indicating that no hidden cross-peaks or coupling signatures are present.

Additionally, the time-resolved measurements of 2D-IR spectra allow for tracking the dynamic evolution of spectral features. To explore this aspect, spectra were collected at different waiting times (*T*_w_) between pump and probe events (Fig. 6). The shape of the 2D-IR peak exhibits some diagonal broadening that could suggest the presence of two unresolved signals. We analysed the time evolution of this broadening to determine its origin, focusing on the ellipticity of the diagonal signal at different waiting times (0.25, 3, 15, and 45 ps), using the ratio of the full width at half maximum (FWHM) along the diagonal and anti-diagonal directions (Table 2). As the waiting time increased, the ellipticity decreased, with the peaks becoming more rounded and less elongated along the diagonal. This change in lineshape suggests spectral diffusion,^30^ indicating that the broadening observed at *T*_W_ = 0.25 ps was due to an inhomogeneous distribution of structural microstates that do not interconvert on this short timescale, rather than the overlap of two discrete signals. Therefore, this diagonal broadening does not reflect two distinct sub-forms of the ACS active site or the presence of two CO ligands, further confirming the presence of a single CO molecule bound the active site of ACS in a pure A_red_–CO state.

**Table 2:** Two-dimensional lineshape analysis performed on 2D-IR spectra recorded at different waiting times *T*_W_ (see Fig. 6)^a^

Waiting time *T*_W/_ps	FWHM_diagonal_/cm^−1^	FWHM_anti-diagonal_/cm^−1^	Ellipticity
0.25	9.20	3.90	2.36
3	9.00	4.49	2.00
15	8.54	4.74	1.80
45	10.04	6.32	1.59

a Full width at half maximum (FWHM) values along the diagonal and anti-diagonal directions were determined by Gaussian fitting. The ellipticity was calculated as the ratio of FWHM_diagonal_/FWHM_anti-diagonal_. All data were extracted from the 2D-IR signal using pump frequencies in the range of 1980–2010 cm^−1^ and probe frequencies between 1955 and 1980 cm^−1^, corresponding to the 1 → 2 transition. In contrast to the 0 → 1 transition, this signal is not influenced by scattering contributions along the diagonal of the 2D-IR spectrum.

Having established that the nonlinear IR data are fully consistent with the presence of a single CO ligand in the A_red_–CO state under near-native conditions, it is instructive to place these findings in the context of previous spectroscopic studies. In particular, earlier ENDOR experiments suggested the presence of two CO ligands bound to ACS in the A_red_–CO state. A key distinction between the two studies is that our experiments were performed under near-native conditions close to ambient temperature (283 K), whereas the ENDOR measurements were carried out at cryogenic temperature (2 K). This difference in temperature may affect the thermal equilibrium between monocarbonyl and dicarbonyl forms of the A_red_–CO state, the former representing the native form. In addition, the dicarbonyl form was reported upon incubation with a high partial pressure of CO gas, while the present study utilized a milder approach of incubation with CO-saturated buffer. Together, these differences may indicate that the stoichiometry of CO coordination in ACS is dependent on experimental conditions and that a dicarbonyl A_red_–CO state would represents a non-native configuration stabilized under extreme conditions.

## Conclusion

Using nonlinear infrared techniques, we have demonstrated that acetyl-CoA synthase binds a single CO ligand in the A_red_–CO state under near-native conditions. This finding conclusively rules out the two potential scenarios that could lead to a second CO ligand that is invisible to linear infrared spectroscopy: (1) the presence of two uncoupled and chemically indistinguishable CO ligands and (2) the possibility that one of the two ligands remains undetected due to a significantly lower transition dipole moment in one of the two CO stretch modes. To systematically address these scenarios, we conducted a detailed examination of the IR_pump_–IR_probe_ spectral profiles, including anharmonicity extraction, lineshape analysis, and evaluation of the spectral time evolution, which unambiguously revealed a single, strongly localized CO stretching mode. In addition, 2D-IR spectroscopy, which is especially sensitive to weakly absorbing but coupled modes and capable of monitoring spectral features over varied timescales, revealed only a single diagonal signal with no cross-peaks or secondary signals, effectively excluding the presence of a low-dipole, hidden ligand. Moreover, anharmonic frequency calculations revealed that a dicarbonyl configuration would produce distinct spectral signatures, even if the second CO was not metal-bound, such as altered anharmonicities that are inconsistent with our experimental observations from both nonlinear infrared techniques used.

By establishing a single-CO configuration under near-native conditions, our study refines the mechanistic understanding of CO coordination in acetyl-CoA synthase. Specifically, our data indicate that CO inhibition observed for monofunctional acetyl-CoA synthase from *Carboxidothermus hydrogenoformans* is not due to a Ni-dicarbonyl state, consistent with other studies.^50,51^ A putative dicarbonyl form of the A_red_–CO state may not be an adequate model of the proposed ‘two-substrate-bound’ catalytic intermediate of acetyl-CoA synthase. Our results also indicate that the native enzyme is not suited for catalysing the formation of C2 compounds from two coordinated CO molecules. It will be interesting to see whether such an activity can be achieved by modification of the active site alcove or the adoption of non-native reaction conditions.

In a wider sense, the experimental and computational strategies employed here can be adapted to explore ligand binding and stoichiometry of other complex organometallic systems in biology and beyond.

## Experimental and computational procedures

### General manipulation

All expression, purification steps, and sample preparations were carried out in a model B anaerobic chamber from COY Laboratory (Michigan, USA). The atmosphere was 95% N_2_ and 5% H_2_, containing 10 ppm of oxygen. To prevent unwanted metals like Zn^2+^ from binding to the active site of ACS, all glass ware was thoroughly washed with 37% v/v HCl and double-deionized water, and buffers were supplemented with 5% w/v Chelex 100 resin (Bio-Rad). Solutions were made anoxic by at least seven cycles of vacuum and nitrogen gassing at a gas train.

### Protein expression and purification

We expressed recombinant ACS from *Carboxydothermus hydrogenoformans* (cloned in a pQE30 vector with a *N*-terminal twin strep-tag) in *Escherichia coli* M15 pREP4 at 37 °C in a terrific broth (TB) medium under nitrogen flow. The medium was supplemented with 40 mM sodium fumarate, 0.5 mM cysteine, 0.1 mM FeSO_4_·7H_2_O, and 1% w/v glucose, and the selection was done with carbenicillin and kanamycin. Expression was induced with 0.5 mM isopropyl-β-*d*-1-thiogalactopyranoside (IPTG) at an OD of 0.8–1.0 at 600 nm. The temperature was set to 30 °C, and the cells were harvested after 20 h. If not used immediately, the cells were frozen in liquid nitrogen and stored at −80 °C. Cells were resuspended in Buffer A (50 mM Tris/HCl pH 8, 100 mM NaCl, 2 mM TCEP; 5 mL buffer per g per cells) supplemented with trace amounts of lysozyme, DNaseI, and Avidin, and incubated for one hour. Afterwards, the cell suspension was sonicated two times for 5 minutes in a rosette cell on ice and ultracentrifuged in gas-tight tubes at 12 °C at 35 000 rpm for 45 min. The soluble fraction was loaded onto a 5 mL Strep-Tactin XT4F column (IBA) previously washed with buffer A supplemented with 2 mM sodium dithionite to purge oxygen and then equilibrated in buffer A. ACS was eluted with buffer A containing 50 mM Biotin, and concentrated with a 50 kDa Amicon ultra centrifugal filter. The protein concentration was determined using a molar extinction coefficient of 134.6 mM^−1^ cm^−1^ (280 nm). The *N*-terminal twin strep-tag was removed by overnight incubation with TEV (Tobacco etch virus) protease in a ratio of 1 : 100 in the presence of 10 mM mercaptoethanol. The mixture was applied to a 3 mL Strep-Tactin XT4F column previously purged from oxygen and equilibrated with buffer A; ACS was eluted in the flow-through fraction, and concentrated as above. The purity of the sample was over 95% as judged by sodium dodecyl sulfate polyamide gel electrophoresis (SDS-PAGE). Nickel reconstitution was performed with a six-fold molar excess of NiCl_2_ in the presence of 10 mM mercaptoethanol at 45 °C, 300 rpm for 75 h. Reconstituted ACS was desalted by gel filtration with a NAP25 column (Cytiva, Massachusetts, USA) equilibrated in buffer A. ACS was concentrated to 0.6 mM, frozen and stored in liquid nitrogen.

### Preparation of the A_red_–CO state

We prepared the samples by reducing ACS with a CO-saturated Ti(iii)-EDTA buffer (0.1 M MOPS pH 7.2) in a 1 : 10 ratio. The Ti(iii)-EDTA buffer was prepared as described previously.^52^ The reduced ACS samples (incubation time 20–30 minutes) were concentrated up to 1 mM, aliquoted to 20–30 µL, frozen and stored in liquid nitrogen.

### IR spectroscopy

Inside a N_2_-filled glovebox, the Ti(iii)-EDTA-reduced and CO-incubated protein solution of ACS (0.3 mM) was injected into a gas-tight IR transmission cell containing two sandwiched CaF_2_ windows and a PTFE spacer (50 µm path length). The cell was transferred to a vertex 80 v/s FTIR spectrometer, equipped with a liquid nitrogen-cooled mercury-cadmium-telluride (MCT) detector. IR spectra were recorded at 283 K with a spectral resolution of 2 cm^−1^. The Bruker OPUS software, version 6.5 or higher, was used for data acquisition and interpretation.

### EPR spectroscopy

The EPR sample of Ti(iii)-EDTA-reduced and CO-incubated protein solution (0.3 mM and 100 µL) was precooled in cold ethanol (*ca.* 200 K) and afterwards flash-frozen in liquid nitrogen, where the sample was stored until the measurement. The EPR spectrum was recorded on a Brucker EMXplus spectrometer equipped with an ER4122 SHQE resonator and an Oxford EPR 900 helium-flow cryostat with temperature adjustment by an Oxford ITC4 controller. The background from the resonator was corrected by subtracting a buffer spectrum from the spectrum of the protein solution measured with the same experimental parameters. Broad drifts of the baseline were additionally corrected using a spline function. Experimental parameters: microwave power = 1 mW, microwave frequency = 9.29 GHz, modulation amplitude = 10 G, and modulation frequency = 100 kHz.

### Ultrafast IR_pump_–IR_probe_ and 2D-IR spectroscopy

The Ti(iii)-EDTA-reduced and CO-incubated protein solution of ACS (1 mM) was handled as described for linear IR experiments. All nonlinear IR spectra were recorded in transmission mode using a gas-tight and temperature-controlled (*T* = 283 K) small-volume sandwich cell (optical path length = 50 µm, *V* ≈ 8 µL) equipped with CaF_2_ windows. All data were acquired in pump probe geometry utilizing mid-IR pulses (centre frequency = 1950 cm^−1^; bandwidth >300 cm^−1^; pulse duration = 50 fs; repetition rate = 10 kHz) from the ULTRA laser system as described previously.^26–29^ Series of IR_pump_–IR_probe_ spectra (accumulation time = 3 s) were recorded by scanning the pump–probe delay time from −14 to 71 ps (step size = 250 fs).

2D-IR spectra (accumulation time = 300 s) were obtained at several selected waiting times *T*_W_ between 250 fs and 45 ps. For each fixed *T*_W_, 2D-IR data were obtained in a time-domain fashion by scanning the coherence time *τ* between two pulse-shaper generated collinear pump pulses from 0 to 6 ps (step size 30 fs) prior to overlap with the probe pulse and self-heterodyned detection of the collinearly emitted signal.

The pump frequency axis was obtained by Fourier transformation of the time-domain signal with respect to *τ*, while the probe frequency axis of both 2D-IR and IR_pump_–IR_probe_ spectra was obtained by signal dispersion in two spectrographs and detection *via* two liquid-nitrogen cooled 128-element MCT detectors (spectral resolution <2.5 cm^−1^). Four-frame phase cycling was employed in 2D-IR data acquisition to limit contributions from pump light scattered on the detector.

## Quantum chemical calculations

All quantum mechanical calculations were performed using Gaussian16 (revision A.03).^53^ The BP86 functional^54,55^ was employed throughout, with the def2-tzvp basis set^56^ applied to the nickel ions (Ni_p_ and Ni_d_) and the 6–31 g(d) basis set^57,58^ for all other atoms.

The methodology used for the anharmonic frequency calculations follows established protocols that have been successfully applied to related systems.^46,59^ Throughout all calculations, we used a ‘superfine’ pruned integration grid for computing two-electron integrals and their derivatives (175/250 radial shells for first-/higher-row atoms, with 974 angular points per shell). SCF convergence criteria were set to 10^−9^ (RMS change) and 10^−7^ (maximum change) in the density matrix, corresponding to an energy change threshold of 10^−18^ hartree.

The computational models were designed to represent the A_red_–CO state of the ACS A-cluster, focusing on the first coordination sphere of the nickel ions (Ni_p_ and Ni_d_). The models were derived from previously established crystallographic structures (PDB ID: 7NYS, DOI: 10.2210/pdb7NYS/pdb). To reduce computational cost associated with anharmonic frequency calculations, the [4Fe4S] cluster was not included in the models. Of note, the current study focusses on the discussion of diagonal and off-diagonal anharmonicities. In case of a mononuclear carbonyl motif, these quantities are determined by fundamental CO bond properties and – in case of the hypothetic dicarbonyl scenario – the bonding interactions between the two CO ligands. As a consequence, the anharmonicities represent locally defined quantities with little sensitivity towards outer coordination shells of the carbonylated metal ion. To illustrate this aspect, key properties of single-CO computational models with and without the [4Fe4S] cluster are compared in Tables S9–S11.

Three computational models were developed to investigate possible CO binding scenarios. The first model A represents the canonical A_red_–CO state, featuring a single CO molecule bound axially to the Ni_p_^1+^ site. The second model B incorporates an additional CO molecule, proposed to bind equatorially at the Ni_p_ site, based on recent ENDOR/EPR findings.^7^ In this configuration, the second CO is positioned to mimic the binding site typically occupied by the methyl group during catalysis. The third model C contained a second, non-coordinated CO ligand close to Ni_p_. For models A and B, the terminal cysteine on Ni_p_ was modelled as anionic (deprotonated). For hypothetical model C, the terminal cysteine coordinated to Ni_p_ was modelled as a neutral (protonated) since no geometry convergence was observed for a model employing an anionic cysteine in that particular case. For models A and B, the impact of terminal-cysteine protonation was found to be negligible. All models underwent full geometry optimization using an analytical Hessian matrix in the final optimization steps, applying stringent convergence criteria to ensure structural accuracy. These criteria included a maximum force of 2.0 × 10^−6^, a root-mean-square (RMS) force of 1.0 × 10^−6^, a maximum displacement of 6.0 × 10^−6^, and an RMS displacement of 4.0 × 10^−6^ (all in atomic units), providing reliable structures for subsequent vibrational analyses.

Harmonic vibrational frequencies were calculated *via* diagonalization of the mass-weighted Hessian matrix. To capture anharmonic effects, Generalized Second-Order Vibrational Perturbation Theory (GVPT2)^31–34^ was applied to compute the anharmonic vibrational transitions, including fundamentals, overtones, and combination transitions.

## Author contributions

Denise Poire: data curation, formal analysis, investigation, methodology, software, validation, visualization, writing – original draft, writing – review & editing; Cornelius C. M. Bernitzky: investigation, writing – review & editing; Mathesh Vaithiyanathan: investigation, writing – review & editing; Berta M. Martins: investigation, writing – review & editing; Christian Lorent: investigation, writing – review & editing; Tamanna M. Ahamad: investigation, writing – review & editing; Vladimir Pelmenschikov: investigation, writing – review & editing; Igor Sazanovich: methodology, writing – review & editing; Gregory M. Greetham: methodology, software, writing – review & editing; Ingo Zebger: funding acquisition, resources, supervision, writing – review & editing; Holger Dobbek: funding acquisition, resources, writing – review & editing; Maria Andrea Mroginski: funding acquisition, resources, supervision, writing – review & editing; Marius Horch: conceptualization, data curation, formal analysis, funding acquisition, investigation, methodology, project administration, supervision, validation, visualization, writing – original draft, writing – review & editing.

## Conflicts of interest

There are no conflicts to declare.

## Supplementary Material

SC-OLF-D5SC08875E-s001

## Data Availability

The data supporting this article have been included as part of the supplementary information (SI). Supplementary information is available. See DOI: https://doi.org/10.1039/d5sc08875e.
