# Supplementary material for: From two to one: resolving CO binding in acetyl-CoA synthase
Source: Chem Sci. 2026 Jan 29;17(13):6755–65. doi: 10.1039/d5sc08875e (PMC12893122; doi:10.1039/d5sc08875e)
Supplement: SC-017-D5SC08875E-s001 [file SC-017-D5SC08875E-s001.pdf]

Supplementary Information (SI) for Chemical Science.
This journal is © The Royal Society of Chemistry 2026

Supplementary Information

From Two to One: Resolving CO Binding in Acetyl-CoA Synthase

Denise Poire,[a,b] Cornelius C. M. Bernitzky,[a] Mathesh Vaithiyanathan,[a] Berta M. Martins,[c] Christian Lorent,[d] Tamanna M. Ahamad,[d] Vladimir Pelmenschikov,[b] Igor V. Sazanovich,[e] Gregory M. Greetham,[e] Ingo Zebger,[d] Holger Dobbek,[c] Maria Andrea Mroginski,[b] Marius Horch*[a]

[a] Freie Universität Berlin, Department of Physics, Ultrafast Dynamics in Catalysis, Arnimallee 14, 14195 Berlin, Germany. E-mail: marius.horch@fu-berlin.de

[b] Technische Universität Berlin, Department of Chemistry, Modelling of Biomolecular Systems, Straße des 17. Juni 135, 10623 Berlin, Germany

[c] Humboldt-Universität zu Berlin, Department of Biology, Structural Biology / Biochemistry, Unter den Linden 6, 10099 Berlin, Germany

[d] Technische Universität Berlin, Department of Chemistry, Spectroscopic Characterization of Metalloproteins, Straße des 17. Juni 135, 10623 Berlin, Germany

[e] STFC Central Laser Facility, Research Complex at Harwell, Rutherford Appleton Laboratory, Harwell Campus, Didcot, OX11 0QX, UK

Contents

Supplementary Figures S1–S9 p. S1

Supplementary Tables S1–S11 p. S7

Supplementary Figures (all data correspond to ACS from *C. hydrogenoformans* in the A_{red}-CO state)

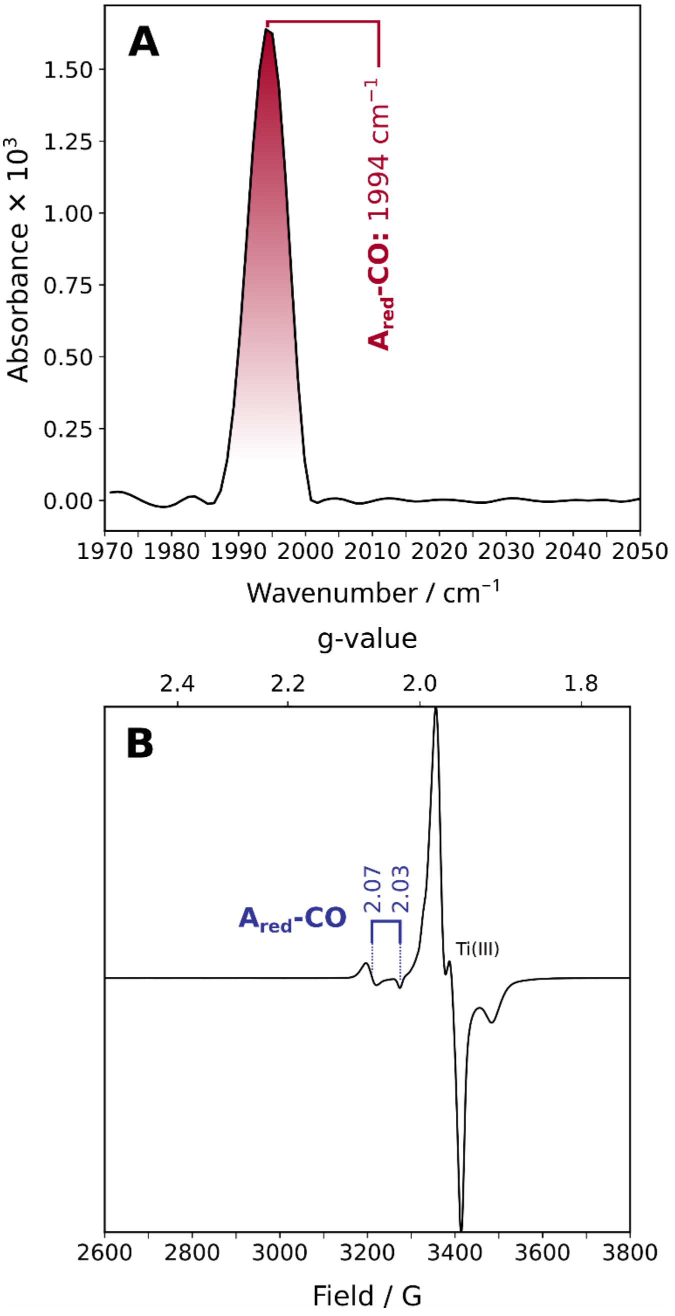

Figure S1: Linear IR absorption spectrum (A) and X-band EPR spectrum (B) of the A_{red}-CO state of ACS from *C. hydrogenoformans*. The IR spectrum was recorded at 283 K, and the EPR spectrum was recorded at 80 K. Both plots depict an extended range of the data shown in Figure 2 of the manuscript.

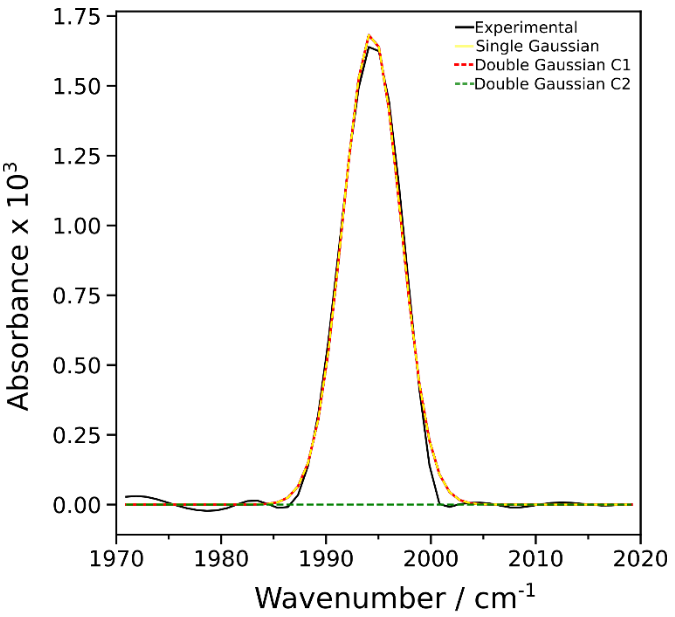

Figure S2: Gaussian fitting of the linear IR absorption spectrum. The experimental IR absorption spectrum is shown in black and the single Gaussian fit in yellow. The individual components of the double Gaussian fit are shown as dashed red (Component 1; overlapping with the single Gaussian) and dashed green (Component 2) lines.

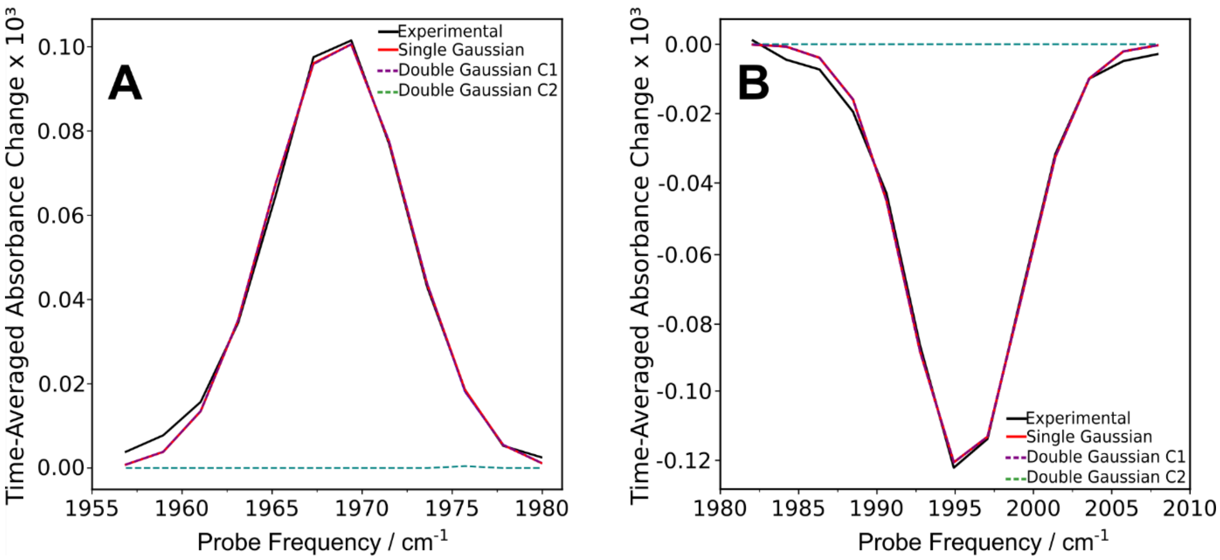

Figure S3: Gaussian fitting of IR_{pump}-IR_{probe} spectra, averaged over all delay times, reflecting (A) the spectral region corresponding to the 1→2 transition and (B) the region of the 0→1 transition. The experimental data is shown in black and the single Gaussian fit in red. The individual components of the double Gaussian fit are shown as dashed purple (Component 1; overlapping with the single Gaussian) and dashed green (Component 2) lines. Fit metrics and parameter estimates are summarized in Table S2 (0→1) and Table S3 (1→2).

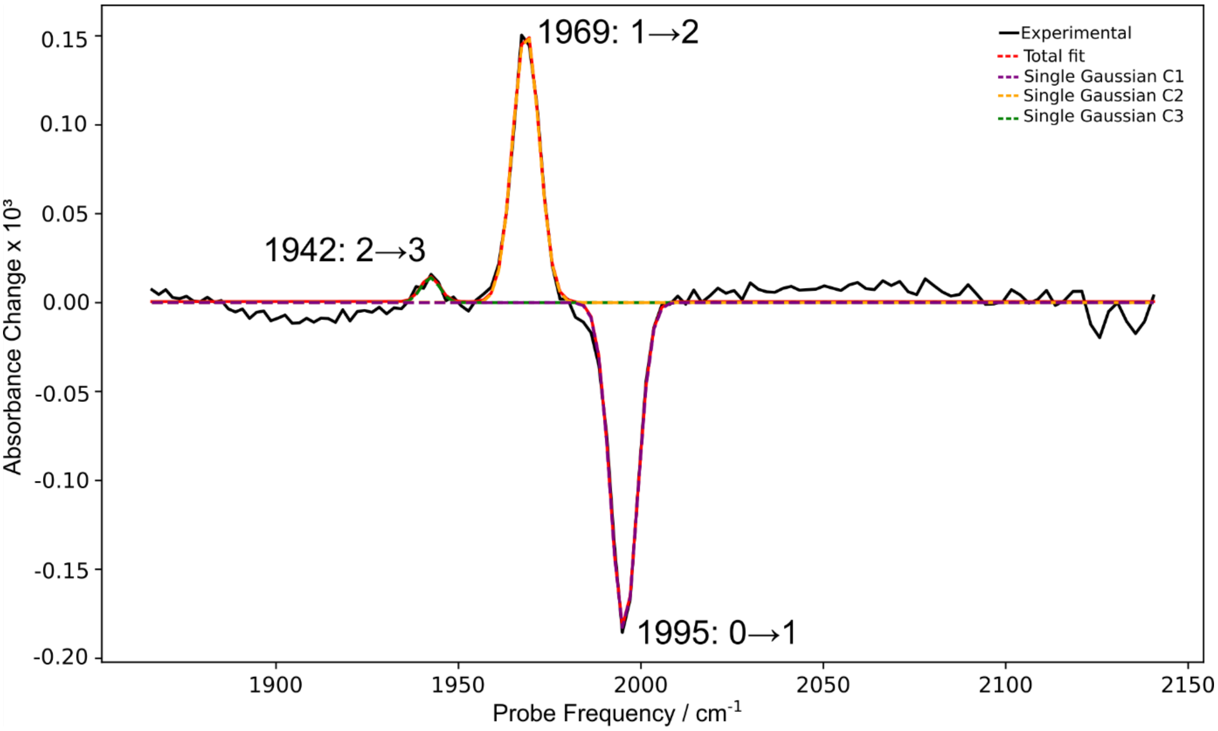

Figure S4: Determination of anharmonicity values from the IR_{pump}-IR_{probe} spectrum recorded at a pump-probe delay time of 0.6 ps with parallel pump-probe polarization. The sum of three Gaussian lineshape functions was fitted to the experimental data to extract the peak positions of the 0→1, 1→2, and 2→3 transitions. The experimental data is shown in black, while the fitted sum of three Gaussian lines is represented in dashed red. The individual Gaussian components are shown as dashed purple (0→1), dashed yellow (1→2), and dashed green (2→3) lines. The extracted peak positions and the corresponding anharmonicity values are summarized in Table S4.

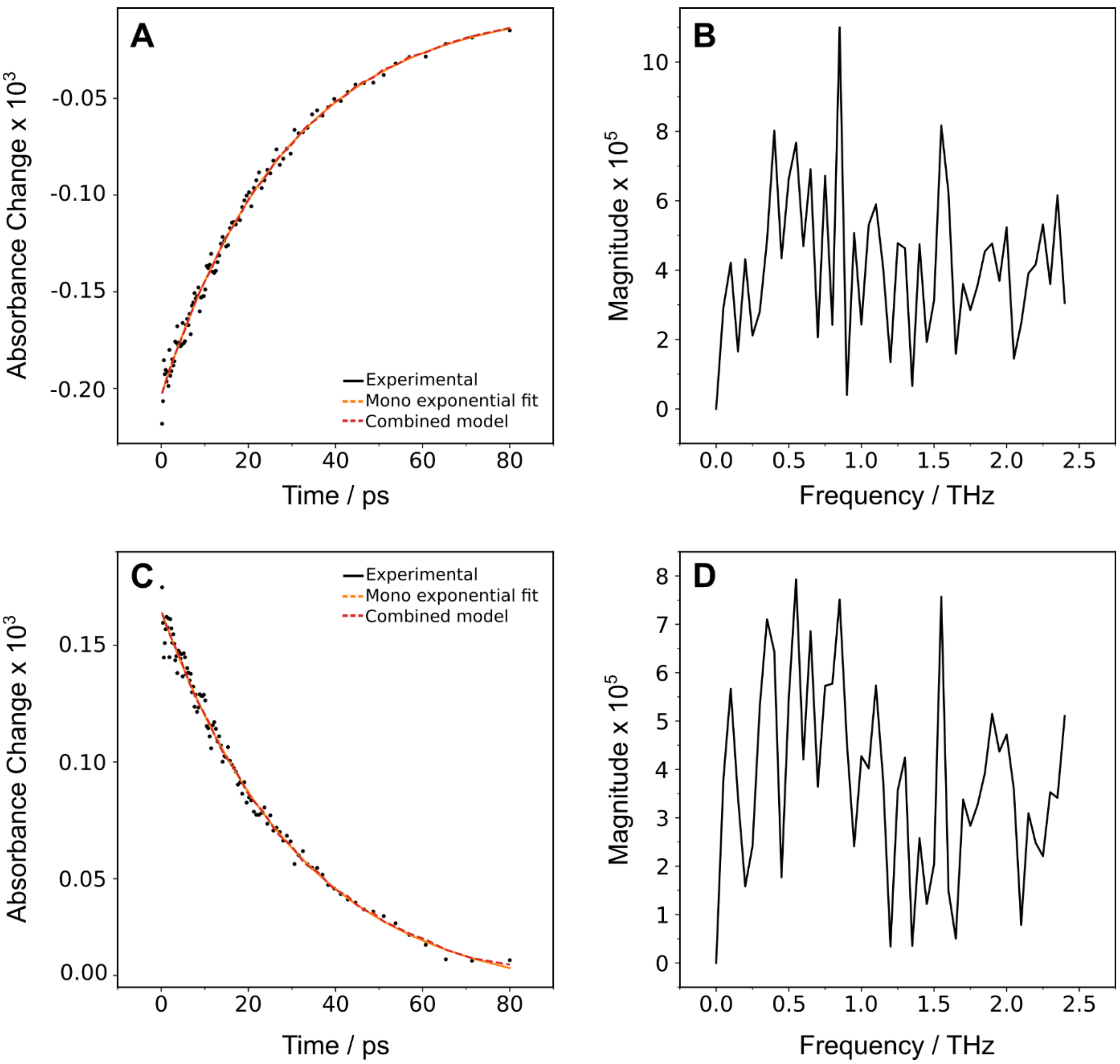

Figure S5: Analysis of time traces from IR_{pump}-IR_{probe} spectra. (A, C) Monoexponential and combined (monoexponential + exponentially damped sine) fit models for time traces obtained at (A) 1969 cm^{-1} and (C) 1995 cm^{-1}. Experimental data are shown in black, the monoexponential fit in orange, and the combined model in yellow. (B, D) Corresponding 'quantum beat' spectra obtained by Fourier transformation of the residuals of the monoexponential fits to the time traces at (B) 1969 cm^{-1} and (D) 1995 cm^{-1}. The residuals represent the part of the time traces that is not captured by the monoexponential model, i.e. any oscillatory component would be contained in the residuals and reflected by their Fourier transform spectra. Fit metrics are summarized in Table S7.

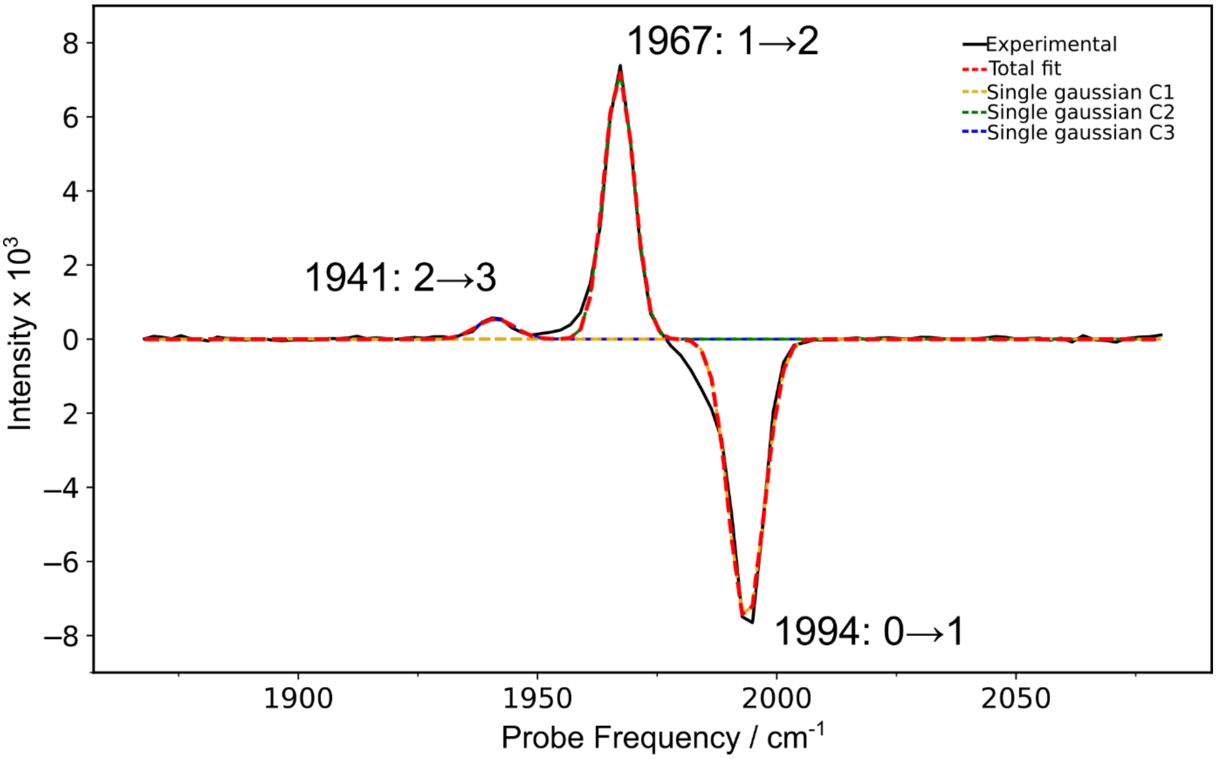

Figure S6: Gaussian fitting of a horizontal through the 2D-IR spectrum obtained at a pump frequency of 1994 cm^{-1}. The experimental data (black) and the sum of three Gaussian components used for fitting (dashed red) are shown. This analysis was used to extract the peak positions of the 0→1, 1→2, and 2→3 transitions. The individual Gaussian components are shown as dashed yellow (Component 1), dashed green (Component 2), and dashed blue (Component 3). The extracted peak positions and the corresponding anharmonicity values are summarized in Table S8. The 2D-IR spectrum was recorded with parallel polarization of pump and probe pulses.

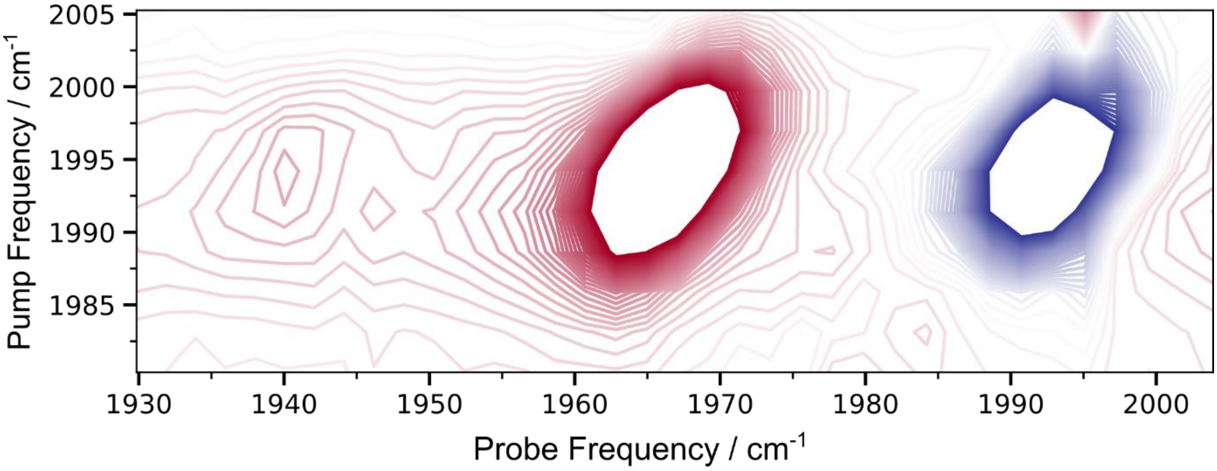

Figure S7: 2D-IR spectrum recorded at T_W = 0.250 ps. The data is identical to those shown in Figure 6, but the spectrum is displayed with an increased number of contour levels (60 instead of 23) to enhance the visibility of low-intensity features. The 2D-IR spectrum was recorded with parallel polarization of pump and probe pulses.

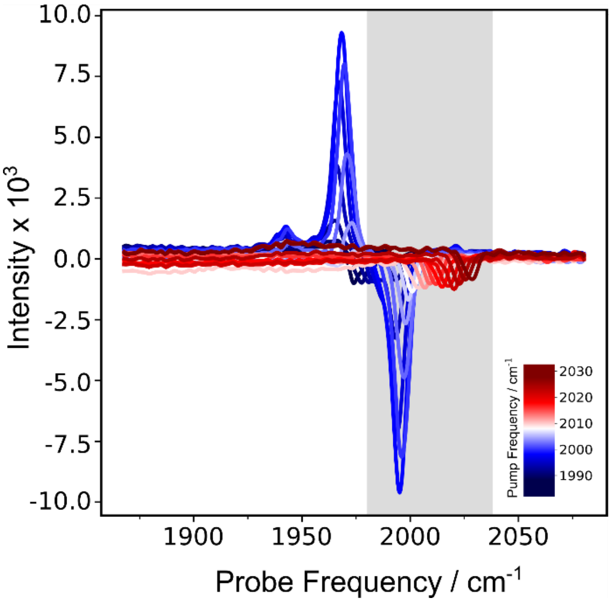

Figure S8: Horizontal slices through the 2D-IR spectrum at pump frequencies from 1980 to 2038 cm^{-1}. The spectra were recorded at T = 283 and T_W = 0.250 ps with parallel polarization of pump and probe pulses. The data correspond to those shown in Figure 6. The pump range is indicated by a light grey layer.

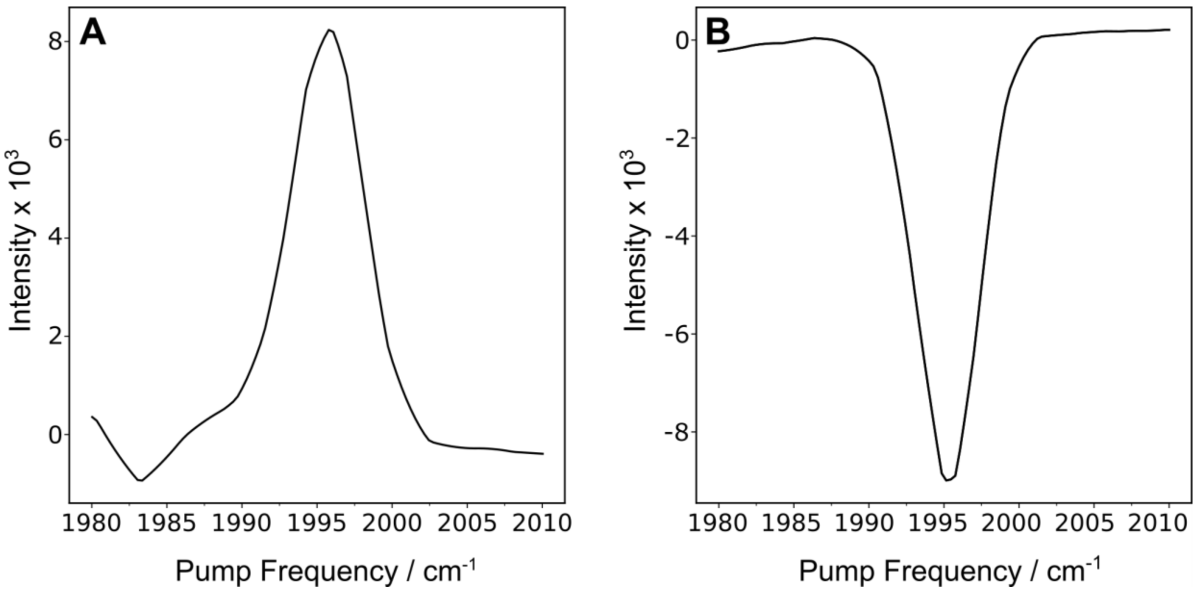

Figure S9: Antidiagonal slices taken through the peak maxima of the 2DIR spectrum (pump maximum ≈ 1994 cm^{-1}, pump range 1980–2010 cm^{-1}). (A) Slice through the 1→2 transition, extracted with probe frequencies 1955–1980 cm^{-1} (B) Slice for the 0→1 transition, extracted with probe frequencies 1980–2010 cm^{-1}. The spectrum was recorded at T = 283 K and T_W = 0.250 ps with parallel polarization of pump and probe pulses. The data correspond to those shown in Figure 6. The dip at ca. 1983 cm^{-1} in Figure S9A corresponds to the point where the antidiagonal slice cuts though the diagonal of the 2D-IR spectrum. The observed bleach reflects pump light scattering along the diagonal.

Supplementary Tables (all data correspond to ACS from *C. hydrogenoformans* in the A_{red}-CO state)

Table S1: Fit metrics and parameter estimates from the IR absorption spectrum using single (G_1) and double (G_2) Gaussian models.‡

**Model**	**AIC**	**BIC**	A_1	ω_1	σ_1	A_2	ω_2	σ_2	R^2
G_1	-739.59	-734.84	0.001684	1994.31	2.77	–	–	–	0.9965
G_2	-733.59	-724.09	0.001684	1994.31	2.77	0.000001	2006.31	0.50	0.9973

‡AIC: Akaike Information Criterion. BIC: Bayesian Information Criterion. A_1, A_2: Amplitudes of the first and second Gaussian components. ω_1, ω_2: Center frequencies of the Gaussian components (cm^{-1}). σ_1, σ_2: Standard deviations of the Gaussian components. R^2: Coefficient of determination.

Table S2: Fit metrics and parameter estimates obtained by applying single (G_1) and double (G_2) Gaussian models to the 0→1 transition in the time-averaged IR_{pump}-IR_{probe} spectra (see Figure S2).‡

**Model**	**AIC**	**BIC**	A_1	ω_1	σ_1	A_2	ω_2	σ_2	R^2
G_1	-332.29	-330.60	-0.000123	1995.62	3.54	–	–	–	0.9972
G_2	-326.29	-322.90	-0.000123	1995.62	-3.54	-0.000006	1964.35	-1.66	0.9972

‡AIC: Akaike Information Criterion. BIC: Bayesian Information Criterion. A_1, A_2: Amplitudes of the first and second Gaussian components. ω_1, ω_2: Center frequencies of the Gaussian components (cm^{-1}). σ_1, σ_2 :Standard deviations of the Gaussian components. R^2: Coefficient of determination.

Table S3: Fit metrics and parameter estimates obtained by applying single (G_1) and double (G_2) Gaussian models to the 1→2 transition in the time-averaged IR_{pump}-IR_{probe} spectra (see Figure S2).‡

**Model**	**AIC**	**BIC**	A_1	ω_1	σ_1	A_2	ω_2	σ_2	R^2
G_1	-285.45	-284.25	0.000103	1968.66	3.79	–	–	–	0.9975
G_2	-279.52	-277.13	0.000103	1968.66	3.78	0.000045	1975.83	-0.04	0.9975

‡AIC: Akaike Information Criterion. BIC: Bayesian Information Criterion. A_1, A_2: Amplitudes of the first and second Gaussian components. ω_1, ω_2: Center frequencies of the Gaussian components (cm^{-1}). σ_1, σ_2 :Standard deviations of the Gaussian components. R^2: Coefficient of determination.

Table S4: Center frequencies ω and anharmonicities Δ extracted from the IR_{pump}-IR_{probe} spectrum.‡

Quantity	Value	Error
$\omega_{0\rightarrow1}$	1995.39	±0.11
$\omega_{1\rightarrow2}$	1968.51	±0.13
$\omega_{2\rightarrow3}$	1942.28	±1.19
$\Delta_1 = \omega_{0\rightarrow1} - \omega_{1\rightarrow2}$	26.88	±0.17
$\Delta_2 = \omega_{1\rightarrow2} - \omega_{2\rightarrow3}$	26.23	±1.19

‡All quantities are given in units of cm^{-1}. The error corresponds to the standard deviations of the fitted Gaussian center frequencies, obtained from the covariance matrix of the nonlinear fit.

Table S5: Fit metrics of single (Mono) and double (Bi) exponential fits applied to the IR_{pump}-IR_{probe} decay curves.‡

ω	AIC (Mono)	AIC (Bi)	BIC (Mono)	BIC (Bi)	RSS (Mono)	RSS (Bi)
1969.39	-2441.92	-2441.29	-2434.13	-2428.31	1.81×10^{-09}	1.75×10^{-09}
1994.91	-2467.33	-2464.87	-2459.55	-2451.90	1.40×10^{-09}	1.38×10^{-09}

‡ω: Analyzed probe frequency (cm^{-1}). AIC: Akaike Information Criterion. BIC: Bayesian Information Criterion. RSS: Residual sum of squares for the mono- and biexponential fit.

Table S6: Extracted vibrational lifetimes (T_1) of the first excited vibrational state of the CO stretch mode from monoexponential fits of the IR_{pump}-IR_{probe} decay curves for each vibrational transition.‡

Transition	ω	A	T_1
1→2	1969.39	1.70×10^{-04}	31.88
0→1	1994.91	-2.28×10^{-04}	29.71

‡ω: Analyzed probe frequency (cm^{-1}). A: Fitted amplitude of the monoexponential model. T_1: time constant of the monoexponential decay (ps), representing the vibrational lifetime.

Table S7: Comparison of F-statistics and p-values for a monoexponential decay model versus a damped-sine-modulated exponential model, both applied to the IR_{pump}-IR_{probe} decay curves at 1969 cm^{-1} and 1995 cm^{-1}.‡

**Wavenumber (cm^{-1})**	**F-statistic**	**p-value**	**Conclusion**
1969.39	0.6360	0.53167	No significant improvement ($p \geq 0.05$)
1994.91	0.8757	0.41995	No significant improvement ($p \geq 0.05$)

‡F-statistic: Ratio of variances used in the F-test to compare two nested models. P-value: Probability of obtaining an F-statistic at least as extreme under the null hypothesis.

Table S8: Center frequencies ω and anharmonicities Δ extracted from the 2D-IR spectrum.‡

**Quantity**	**Value**	**Error**
$\omega_{0\rightarrow1}$	1993.65	± 0.09
$\omega_{1\rightarrow2}$	1967.00	± 0.08
$\omega_{2\rightarrow3}$	1941.15	± 1.21
$\Delta_1 = \omega_{0\rightarrow1} - \omega_{1\rightarrow2}$	26.65	± 0.12
$\Delta_2 = \omega_{1\rightarrow2} - \omega_{2\rightarrow3}$	25.85	± 1.21

‡All quantities are given in units of cm^{-1}. The error corresponds to the standard deviations of the fitted Gaussian center frequencies, obtained from the covariance matrix of the nonlinear fit.

Table S9: Spin densities and atomic charges for the single-CO model with and without the [4Fe-4S] cluster.

Atom	Mulliken charge: small model	Mulliken charge: large model	Spin density: small model	Spin density :large model	NPA charge: small model	NPA charge: large model
Ni_p	-0.356739	-0.408609	+0.404871	+0.406368	-0.51140	-0.400
Ni_d	+0.096489	+0.008753	+0.339935	+0.234571	+0.13629	-0.067
N donor (Gly N1)	-0.419272	-0.407431	+0.031480	+0.008949	-0.55261	-0.28687
N donor (Gly N2)	-0.420658	-0.409108	+0.038428	+0.011620	-0.54864	-0.28773
S donor (Cys S1)	+0.044915	+0.164477	+0.081975	+0.081931	+0.06139	+0.02527
S donor (Cys S2)	+0.050158	+0.084410	+0.050985	+0.024482	+0.06474	+0.01966
S donor (bridging Cys)	+0.009312	+0.130652	+0.054583	+0.014018	+0.15295	+0.07916
CO carbon	+0.279416	+0.312370	-0.023422	-0.011051	+0.53181	+0.26875
CO oxygen	-0.302920	-0.342617	-0.013504	-0.011436	-0.48701	-0.25388

Table S10: Distances for the single-CO model with and without the [4Fe-4S] cluster.

Bond	**Bond length (Å):** small model	Bond length (Å): large model
Ni_p–CO	1.77	1.76
C–O	1.17	1.18
Ni_p–Ni_d	2.88	2.60
Ni_d–N (Gly N1)	2.12	1.93
Ni_d–N (Gly N2)	2.01	1.88
Ni_d–S (Cys S1)	2.23	2.25
Ni_d–S (Cys S2)	2.25	2.21
Ni_p–S (Cys S1)	2.37	2.29
Ni_p–S (Cys S2)	2.33	2.32
Nip–S (bridging cysteine)	2.30	2.35

Table S11: Intramode anharmonicity of the CO stretching mode for the single-CO models with and without the [4Fe-4S] cluster.

Model	Intramode anharmonicity / cm^{-1}
Small model	24.618
Large model	24.681
